# Notch receptor–ligand binding and activation: Insights from molecular studies

**DOI:** 10.1016/j.semcdb.2012.01.009

**Published:** 2012-06

**Authors:** Chandramouli R. Chillakuri, Devon Sheppard, Susan M. Lea, Penny A. Handford

**Affiliations:** a Department of Biochemistry, University of Oxford, South Parks Road, Oxford, UK; b Sir William Dunn School of Pathology, University of Oxford, South Parks Road, Oxford, UK

**Keywords:** EGF, epidermal growth factor, CSL, CBF1/suppressor of hairless/Lag1, Notch signalling, cis/trans, Notch, Jagged, Epidermal growth factor-like

## Abstract

The Notch receptor is part of a core signalling pathway which is highly conserved in all metazoan species. It is required for various cell fate decisions at multiple stages of development and in the adult organism, with dysregulation of the pathway associated with genetic and acquired diseases including cancer. Although cellular and *in vivo* studies have provided considerable insight into the downstream consequences of Notch signalling, relatively little is known about the molecular basis of the receptor/ligand interaction and initial stages of activation. Recent advances in structure determination of the extracellular regions of human Notch-1 and one of its ligands Jagged-1 have given new insights into docking events occurring at the cell surface which may facilitate the development of new highly specific therapies. We review the structural data available for receptor and ligands and identify the challenges ahead.

## Highlights

► We review the high resolution structures of the Notch receptor and ligands. ► Highlight the docking events of Notch receptor and ligand at the cell surface. ► Indicate the future challenges in understanding Notch receptor–ligand interactions.

## 1 Introduction

**Fig. 1:**
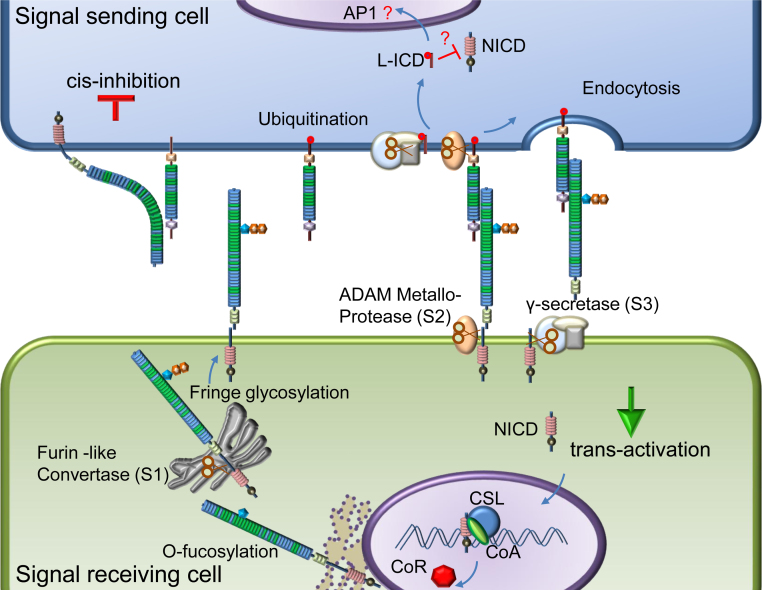
Canonical Notch signalling. The Notch receptor undergoes post-translational modifications, including glycosylation and proteolysis (S1) before being targeted to the cell membrane. A ligand from the neighbouring cell binds to the receptor leading to activation. Ubiquitination of ligand and initiation of endocytosis of ligand–receptor complex leads to a second proteolytic cleavage by ADAM metalloprotease (S2) removing the extracellular region. The membrane tethered receptor fragment is cleaved by the γ-secretase complex (S3) to release the Notch intracellular domain (NICD) which translocates into the nucleus. NICD, together with CSL and co-activator Master mind-like (MAML), displaces the co-repressor and forms a transcription activation complex on promoters of target genes like *Hes-1*, *Hes-5* which contain CSL binding sites. Some recent reports show that ligands also undergo proteolysis [50] and release ligand intracellular domain (LICD) which antagonizes Notch signalling by mechanisms as yet unclear. Receptor and ligand present on the same cell surface can also bind to each other leading to cis-inhibition. The molecular basis of the cis-inhibitory complex is unknown, but implicates similar regions of receptor and ligand to those involved in trans-activation.

The Notch receptor is a single pass trans-membrane protein which, during maturation, may be cleaved by a furin-like convertase (at S1) in the trans-Golgi to generate a non-covalently associated heterodimer at the cell surface. Canonical Notch signalling is initiated when a cell-surface expressed Delta/Serrate/LAG-2 (DSL) ligand binds to the Notch receptor expressed on an opposing cell surface (Fig. 1). Endocytosis of the Notch-ligand complex by the ligand-expressing cell leads to ADAM metalloprotease mediated cleavage at S2 and removes the extracellular fragment of the heterodimer. The membrane tethered fragment is then cleaved by γ-secretase complex at S3 to release the intracellular fragment of Notch (NICD). This translocates to the nucleus and assembles into a transcriptional activation complex which includes a DNA binding protein of the CSL family and its co-activator Mastermind-like. This new assembly relieves repression of Notch target genes such as *Hes-1*. In addition to trans-activating Notch–ligand complexes, the receptor can also form cis-inhibitory complexes (Fig. 1) when Notch and ligand are expressed on the same cell surface. Cis-inhibition serves to limit the zone of Notch activity and is particularly important in developmental programs in *Drosophila* such as the wing disc and eye.

## 2 Domain architecture of Notch receptor

**Fig. 2:**
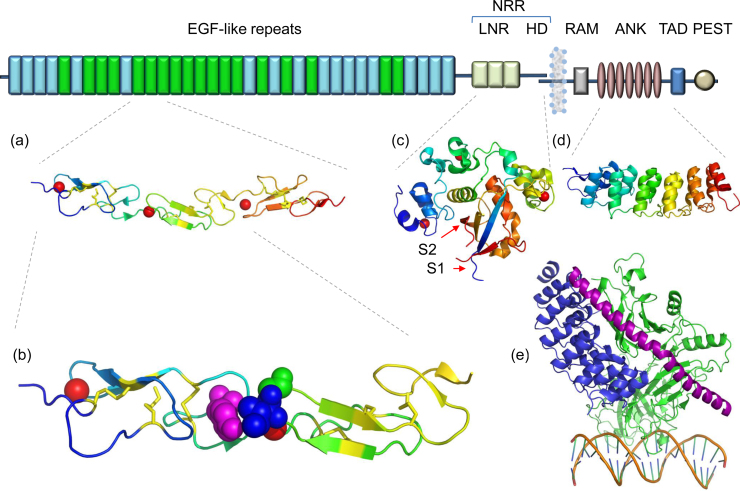
Architecture of Notch1 receptor. Human Notch-1 is represented with major domains annotated. In the EGF repeat region the Ca^2+^ binding EGF domain is green and non-Ca^2+^ binding EGF domain is blue. (a) Crystal structure of EGF11–13 repeats (PDB ID: 2VJ2) which encompass the ligand binding site and show a near linear domain arrangement. (b) Tandem EGF repeats are rigidified by bound Ca^2+^ (red sphere) and interdomain packing of aromatic residues, Tyrosine (purple spheres) from EGF11 with hydrophobic Isoleucine (blue spheres) and Glycine (green spheres) from EGF12. (c) Crystal structure of the negative regulatory region (NRR) (PDB ID: 3I08) comprising three LNR repeats and the heterodimerisation domain shows that the S2 cleavage site is buried deep inside the globular NRR. (d) Crystal structure of ankyrin repeat region ANK (PDB ID: 2F8Y). (e) Transcription activation complex (PDB ID: 2F8X) made up of CSL (green), NICD (blue) and Master mind-like (purple) binding to the CSL binding site (double stranded DNA) on *Hes-1* promoter. RAM peptide region involved in CSL protein binding, trans-activating domain (TAD) and degradation signal PEST are also illustrated in the figure.

The extracellular domain (ECD) of the Notch receptor varies from species to species; *Drosophila* and mammalian receptors are much larger than their counterparts from *Caenorabditis elegans*, although each invariably maintains the same molecular architecture (see review [1]). *Drosophila* has a single Notch receptor, *C. elegans* has two (LIN-12, GLP-1) while mammals have four paralogues. At the N-terminal end, *Drosophila* Notch and human Notch-1 (Fig. 2) both contain 36 EGF-like domains, a subset of which contain calcium-binding sites (cbEGF). Following the EGF-like domains are three Lin-12-Notch (LNR) repeats, and a hydrophobic region which has been shown to mediate heterodimerisation (HD). Together, the LNR repeats and the heterodimerisation domain form the negative regulatory region (NRR), adjacent to the cell membrane. This region prevents ligand-independent activation of the Notch receptor by concealing and protecting the S2 cleavage site from metalloproteases [2]. The S3 cleavage site lies within the transmembrane segment and is cleaved by the γ-secretase complex to liberate NICD. NICD contains a RAM domain, seven ankyrin repeats (ANK), a transcription activation domain (TAD) and PEST domain. Both the RAM domain and ANK repeats have been identified as regions involved in the interaction with CSL transcription factors [3]. The TAD region is found in Notch-1 and -2 but not in -3 and -4 in mammals. The C-terminal PEST domain is involved in NICD degradation by proteolysis. Mutations which lead to deletions of this region are associated with T-cell leukaemias, emphasising the important functional role of regulated NICD degradation [4].

### 2.1 Structure of the ligand binding domain (LBD) of human Notch1

Deletion analyses, in combination with cell aggregation assays, identified EGF domains 11 and 12 of the *Drosophila* Notch receptor as the major ligand-binding site. This region was found to be sufficient to bind in a calcium-dependent manner to Notch ligands, but it did not show full functionality *in vivo*, indicating that additional sites were involved in Notch activation and regulation [5,6]. The large size and disulphide-bonded complexity of the full length Notch receptor precluded structural studies on the intact native molecule, however analysis of other unrelated EGF domain-rich proteins such as fibrillin-1 demonstrated that a molecular dissection approach employing *in vitro* redox refolding could be used to determine the structure of short fragments containing two or more EGF domains [7,8]. A fragment of human Notch-1 EGF11–13, encompassing the ligand-binding region, was subsequently expressed in bacteria, refolded *in vitro* and shown to be capable of binding to ligand in a Ca^2+^ dependant manner in FACS assay when biotinylated and anchored to Streptavidin beads and also in Surface Plasmon Resonance (SPR) studies [9,10]. A study of calcium-binding mutations introduced into a slightly larger fragment hNotch-1 EGF11–14 showed that the calcium-dependent structure of EGF12 but not EGF11 or EGF13 was key to hDll-1 binding, suggesting that the ligand-binding site resides in Notch-1 EGF12 and/or the EGF11–12 junction. The solution structure of hNotch-1 EGF11–13 showed that EGF11 and 12 packed against each other, with Ca^2+^ dependent pairwise domain interactions stabilised by a conserved aromatic residue (Y/F/W) in the N-terminal domain packing against a hydrophobic residue (I/L/V/P) and a conserved glycine in the C-terminal domain. In contrast, there was a poorly defined orientation for EGF13. The tilt angle of ∼20° for hNotch-1 EGF11–12 was similar to that seen for calcium binding (cb)EGF domain pairs of fibrillin-1, but the twist angle of 120° was very different and at least in part due to the two amino acid linker between EGF11 and 12, rather than the one amino acid linker seen in fibrillin-1. This NMR structure allowed modelling of other contiguous regions of Notch cbEGF domains as rod-shaped structures, since linker length and residues important for pairwise interactions are highly conserved.

A high resolution crystal structure was later solved for an EGF11–13 fragment (Fig. 2a), which showed a calcium-stabilised rod-shape for all three domains with dimensions 100 × 24 × 20 Å [11]. As seen in the NMR structure the interdomain packing interactions were key to maintaining the rigidity of the domain pair (Fig. 2b). The stability of EGF13 in the crystal structure was attributed to the addition of a recombinant C-terminal tag which was absent in the earlier construct used for NMR. The overall elongated shape of this region provides an extensive protein–protein interaction surface which is likely to be of functional significance. Although EGF12 appears to be the core ligand binding domain, it is possible that additional contacts from neighbouring domains may contribute to the interaction. Further work on defining the ligand binding site using site-directed mutagenesis, chemical shift mapping and other interaction studies will help to clarify the binding interface.

O-glycosylation of the extracellular domain is known to play a key role in regulating Notch signalling. Multiple sites occur across the receptor, including one O-fucose site and one O-glucose site in EGF12. Over-expression of Ofut1 (O-fucosyl transferase) in Notch-expressing cells increases Notch-Serrate binding, whereas it decreases the Notch-Delta binding. These results are in contrast to the effects of extending the fucose moiety with N-acetylglucosamine (GlnNAc) by the glycosyltransferase Fringe. This additional modification decreases Notch-Serrate binding and increases Notch-Delta binding [12]. Mutation of the O-fucose site in EGF12 (Ser to Ala) of *Drosophila* Notch eliminates the inhibitory effect of Serrate on Notch receptor because fucosylation and subsequent Fringe modifications do not occur. This mutant was found to be functional during embryonic neurogenesis but not at the dorsoventral boundary. An O-fucose site substitution in EGF12 (Thr to Ala) of mouse Notch1 receptor (Notch1^12f^) demonstrated that homozygous (Notch1^12f/12f^) mice were viable and fertile, though they showed decreased Notch signalling leading to defective T-cell development [13,14]. These data show that post-translational modifications of Notch can modulate Notch ligand interactions, but the relative importance of each site glycosylated and the molecular basis for this regulation is unknown. Recently, controlled *in vitro* glycosylation of synthetic mouse Notch-1 EGF12 was performed and the structure solved by NMR [15]. Only minor structural changes in the domain were observed with the addition of a single fucose residue but addition of a GlcNac residue onto fucose resulted in a significant conformational change in the beta-hairpin of EGF12. Although interesting, the functional significance of these data is not clear. The NMR experiments were performed in the absence of Ca^2+^ on an isolated domain. Since N-terminal linkage of EGF11 and Ca^2+^ are both required for EGF12 to adopt its native ligand-binding structure, further comparative experiments in the presence of Ca^2+^ are required to confirm the structural effects of O-linked modifications to EGF12, and their impact on ligand binding.

### 2.2 Structure of negative regulatory region (NRR) of Notch1

The membrane-proximal NRR, which contains the LNR repeats, the dimerisation domain and both S1 and S2 cleavage sites, is well characterised at an atomic level (Fig. 2c) compared to the EGF repeat region, and has been reviewed in detail recently [16]. Most Notch receptors are cleaved at the S1 site to form a heterodimer molecule, but the requirement of such processing for activity is debatable. A recent report of a S1-cleaved human Notch-1 NRR region shows that there is little change in the overall structure whether or not the S1 loop is present [17]. Several disease-causing mutations associated with T-cell leukaemias have been mapped to the NRR region and have been found to lead to a gain of Notch signalling suggesting that the NRR acts as an activation switch in the receptor. The first NMR structure of the LNR module showed that it has little secondary structure, but is stabilised by three disulphide bonds and a single calcium ion [18]. In an early study calcium was shown to play a key role in stabilising the inactive state of the NRR since EDTA treatment resulted in receptor activation and NICD production in a ligand independent manner [19]. A crystal structure of the NRR region of human Notch-2 showed that the S2 cleavage site is buried because of an extensive interaction surface between the LNR repeats and the heterodimerisation domain (HD), which constitutes two subunits tightly entwined in an α-β-sandwich. As a consequence of the cap-like covering of LNR over the HD region, the protein is locked in an autoinhibited mode. This finding immediately poses the question of how the autoinhibition is relieved and the S2 cleavage site exposed? The current favoured mechanism is that ligand binding and subsequent initiation of ligand endocytosis generates a mechanical pull on the NRR region which leads to LNR repeats being pulled away unmasking the S2 cleavage site (Fig. 1). This is consistent with experimental data which show a requirement for ligand endocytosis in Notch signalling (see review by Weinmaster in this series). However an alternative allosteric mechanism cannot be excluded whereby ligand binding causes a conformational change in the NRR leading to S2 exposure. Irrespective of which of these models is correct, mutations found in T-ALL patients appear to disrupt the hydrophobic core of the HD region leading to exposure of the S2 cleavage site, even in the absence of ligand [20].

### 2.3 Structure of Notch intracellular domain (NICD)

The NICD, encompassing the RAM, ANK and PEST domains is by far the best characterised region in the Notch receptor at the atomic level (for a detailed review see [21]) The ANK domain (Fig. 2d), which comprises seven ANK repeats, was the first region to be studied, probably because of the known classical ANK fold comprising a pair of antiparallel helices, and its observed role in protein–protein interactions [22]. Subsequent biochemical and structural analysis of the Notch transcription complex partners has revealed that the interaction between NICD and CSL is dependant predominantly on the RAM domain and less on the ANK domain [23]. In contrast, the binding of the co-activator MAML1 is independent of RAM domain, but instead requires the ANK domain. It was found that neither NICD nor CSL can bind MAML1 alone, but bind cooperatively to it in a complex (Fig. 2e) [24]. A recent report shows that NICD, CSL and MAML1 cooperatively form a dimer complex on a paired recognition site of a promoter *Hes5* [25]. This finding demonstrates how promoter organisation controls responsiveness to Notch signalling.

### 2.4 Structure of Notch receptor: a rod or a rope

**Fig. 3:**
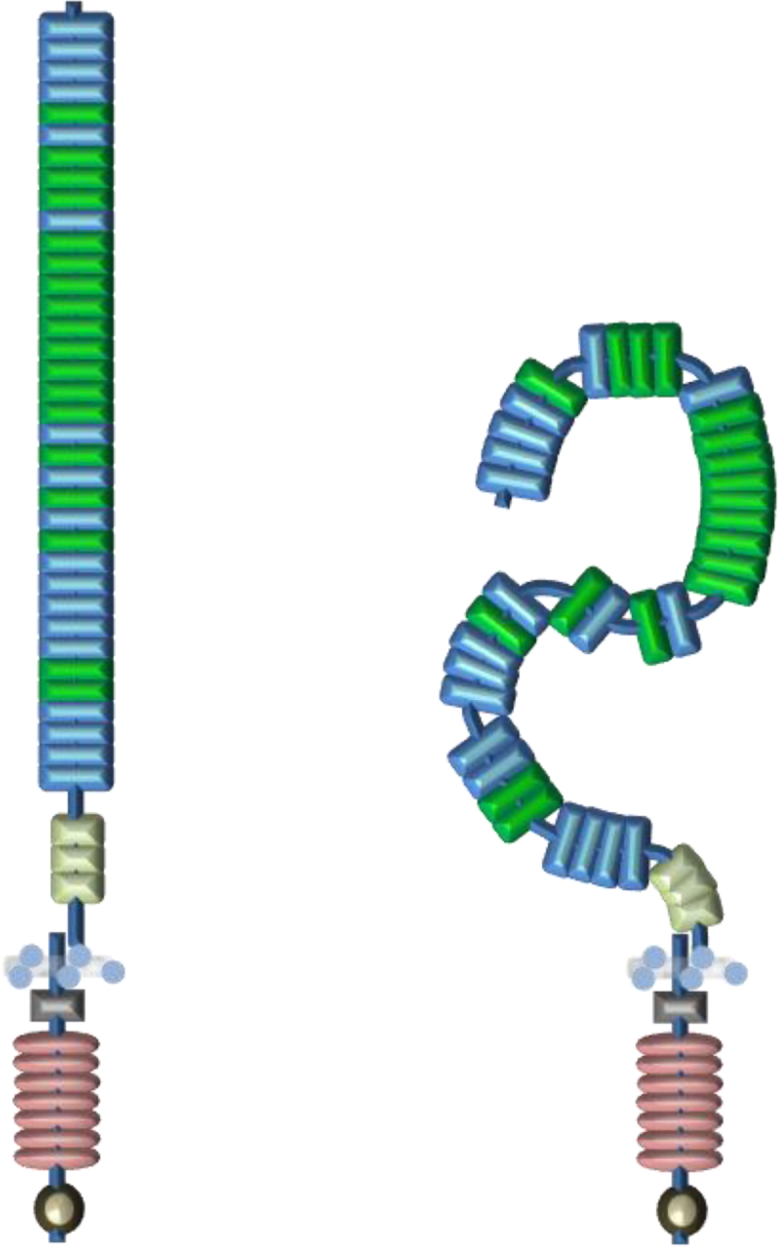
Cell surface organisation of Notch receptor. Much of the extracellular region of the Notch receptor is made up of EGF repeats. The crystal structure of the EGF11–13 region showed that these domains are arranged into a near linear structure rigidified by Ca^2+^. Based on this observation, a near linear model is proposed that extends away from the membrane. There is evidence that the cbEGF–EGF and EGF–EGF tandem domains can be flexible or pack in a non-linear orientation and may adopt a bent rather than a linear structure. Since the N-terminal region and C-terminal region of the extracellular domain (ECD) contains several non Ca^2+^ binding EGF repeats, it is possible that the Notch receptor attains a much more compact structure. It should be noted that the compact structure represented here is showing possible regions of flexibility and is not a structure prediction. If there are any interactions between different regions of the ECD a much more compact structure may exist.

Despite advances in structure determination of limited fragments of Notch comprising the key domain types, the quaternary structure of the ectodomain remains unsolved. Since much of the extracellular region of Notch comprises calcium binding EGF-like domains, it is reasonable to base working models of receptor shape on structural features of this domain type. Most of the receptor is predicted to have a rigid near-linear architecture (Fig. 3), but potential sites of flexibility may occur at the interfaces of cbEGF/EGF and EGF/EGF domains which appear much less conserved [9,26]. An alternative bent structure has been proposed (Fig. 3) based on non-linear pairwise domain interactions, as shown for the EGF pair from a merozoite protein from *Plasmodium falciparum*, which predicts a shorter, more compact, receptor [27]. This model is intriguing in a way that it provides a potential explanation for genetic and biochemical data which implicate domains distant from EGF11–12, such as the *Abruptex* region, in modulating ligand dependent activation. The quaternary structure of the complete extracellular domain (ECD) of human Notch1 receptor and *Drosophila* Notch receptor has recently been investigated by single particle electron microscopy and antibody labelling [28]. These are necessarily challenging experiments as the inherent resolution of the technique is poorly matched to the small size of the domains involved. Baculovirus-expressed proteins were directly captured on grids and dimensions of ∼25 nm estimated for the long axis of each protein. Overall, these dimensions support a more compact rather than extended architecture for the receptor; measurements >100 nm would have been expected for an extended rod-shaped molecule since each EGF domain is ∼3 nm long. Furthermore, the density maps suggested that the ECD existed as a homodimer and could adopt at least three conformations which the authors proposed may be of functional significance to the cis- and trans-regulatory interactions mediated by Notch. However it should be noted that due to low yields of protein, both *Drosophila* and human proteins were directly captured on grids rather than undergoing extensive purification. This could result in inclusion of non-native receptor in the data set. Ideally future single-particle studies should include comparative analyses of purified and characterised proteins, in the presence and absence of Ca^2+^, since Ca^2+^ is known to have substantial effects on the conformation of tandem cbEGF domains. These could be complimented with antibody labelling studies using a panel of monoclonal antibodies specific for individual Notch EGF domains to provide distance restraints in conjunction with structure and dynamics studies of fragments containing cbEGF/EGF linkages.

## 3 Domain architecture of canonical ligands

**Fig. 4:**
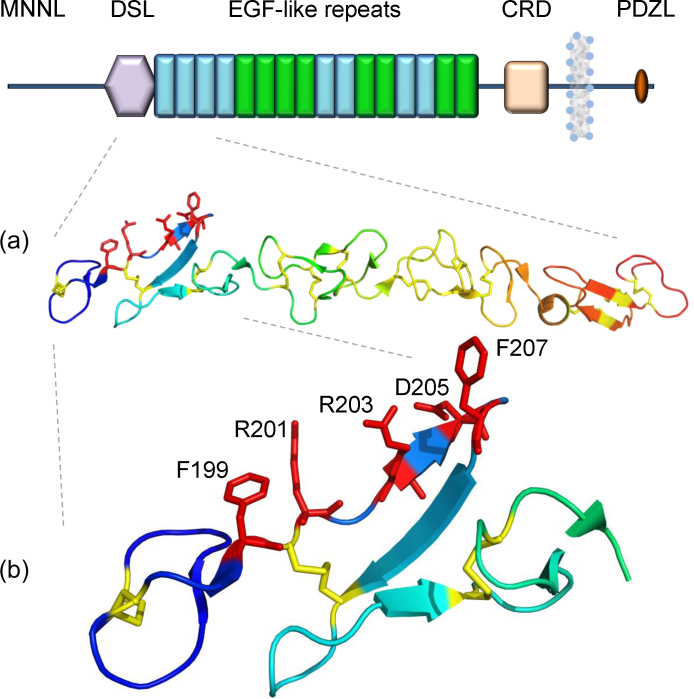
Architecture of Jagged1. Human Jagged-1 is represented in the figure. Similar to the Notch receptor, much of the extracellular region comprises EGF repeats. No structural predictions exist for the N-terminus of Notch ligand (MNNL) region. CRD represents the cysteine-rich region and the PDZL domain is present on the C-terminal region. (a) Crystal structure of human Jagged1 DSL EGF3 region (PDB ID: 2VJ3) showed a near linear arrangement of these domains. Disulphide bonds are shown as yellow sticks (b) The DSL domain is found to have a unique fold and forms the main receptor binding site. Sequence alignments and subsequent mutational studies showed that a subset of residues localised on one face of Jagged1 are crucial for the interactions with the receptor. Side chains of residues F^199^, R^201^, R^203^, D^205^, F^207^ are represented as red sticks.

The Notch receptor is activated by Delta/Delta-like and Serrate/Jagged ligand families (Fig. 4), both of which contain a Notch-binding site within a DSL domain. *Drosophila* contains one member of each family (Delta and Serrate), while mammalian ligands are more complex with three members of the Delta family (Dll-1, -3 and -4) and two members of the Jagged family (J-1 and -2). Preceding the DSL domain is a disulphide-bond stabilised module at the N-terminus of Notch ligands (MNNL) which is of unknown structure, but is functionally important since many missense mutations affecting this region of hJ-1 give rise to Alagille syndrome. Following the DSL domain is a series of EGF-like domains which range in number from 16 (Jagged family), 5–9 (Delta family) and one in DSL-1 (secreted ligand) of *C. elegans* [29]. The first two EGF-like domains in Serrate/Jagged1/2 and Delta/Delta-like-1 are unusual in that, although they contain 6 conserved cysteine residues, they have very short loop sequences and resemble a motif seen in the OSM-11 protein in *C. elegans* known as the Delta/OSM-11 domain (DOS domain) [30]. The remaining EGF domains are more orthodox in sequence. A juxtamembrane cysteine rich domain (CRD) distinguishes Serrate/Jagged from the Delta family. On the cytosolic side, a PDZL domain was identified in some vertebrate ligands (Jagged-1, Delta-like-1, 4). This domain facilitates the interaction with proteins at the adherens junction in promoting cell–cell adhesion and inhibiting cell motility [31].

The crystal structure of a human Jagged-1 fragment containing the DSL domain and the first three EGF-like domains have given the first high resolution insights into ligand domain organisation. The four domains form an elongated structure (Fig. 4a), similar to the ligand binding EGF11–13 region of Notch, with dimensions of 120 × 20 × 20 Å [11]. The DSL domain revealed a unique fold with no known structural homologues, but showed similarity to an EGF domain, since it contained a double-stranded antiparallel β-sheet. The disulphide bond pattern in the DSL domain was found to occur between consecutive cysteine residues instead of the C1–C3, C2–C4, C5–C6 arrangement usually associated with the EGF fold. However, the loop between C1 and C2 superimposed on the loop from C5 and C6 suggesting that the DSL domain might have evolved from a truncation of tandem EGF domains. Sequence alignment of DSL domains from both ligand families identified a group of highly conserved residues (human Jagged1 F^199^, R^201^, R^203^, F^207^) which mapped to one face of the DSL domain forming a putative Notch binding site (Fig. 4b). Functional analysis of site-directed DSL mutants confirmed the importance of these residues in both cis- and trans-regulatory interactions with Notch.

Analysis of the three EGF domains revealed interesting differences. EGF3 showed a classical EGF fold whereas EGF1 and 2 showed a trimmed EGF fold with no canonical secondary structure. These data confirm the observation of Lissemore and Starmer based on sequence alignments that EGF1 and 2 are different from EGF3 [32]. A solution NMR structure of a 44 aminoacid synthetic peptide corresponding to exon6 of human Jagged-1 consisting of the C-terminal region of EGF1 and EGF2 showed that there were strong hydrophobic interactions between Y^255^, W^257^ of EGF1 and I^266^, P^279^, W^280^ of EGF2 indicating the rigidity of these tandem repeats even in solution [33]. Non-canonical EGF domains have been observed in the Delta and OSM-11 (DOS) motif identified in the OSM-11 and related proteins of *C. elegans*, which may act as Notch coligands. The DOS motif comprises of a non-canonical EGF domain and additional sequences including two conserved cysteine residues and a tryptophan residue which may suggest it evolved from two non-canonical EGF domains. However it remains to be confirmed whether it represents a completely novel domain structure with a non-EGF disulphide pairing (as observed in the DSL domain) or comprises a non-canonical EGF domain with an additional disulphide bond.

Further, unresolved questions about ligand structure include the role of the MNNL domain. This region is not conserved between the two ligand families but the number of missense mutations associated with Alagille syndrome which affect amino acids within this domain from Jagged-1 indicate an important functional role which has yet to be elucidated. Similar to the Notch receptor, the cell surface organisation of the ligands remain unsolved. Do the ligands extend away from the cell surface, or adopt a more compact, membrane proximal, architecture? What is the molecular basis for the differential response of each ligand family to O-fucosylation of Notch.

## 4 Notch–ligand interactions

There are two different modes of canonical Notch–ligand interactions. Ligand expressed on the surface of a signal-sending cell can bind in trans to a receptor on the receiving cell initiating Notch signalling [34]. Alternatively ligand can be expressed on the same cell surface as the receptor resulting in a cis inhibitory interaction that limits Notch activity [35]. Although these two modes of interaction are well established from a variety of experimental data, the molecular basis for activating and inhibitory complexes is poorly understood. It has been unambiguously established that Notch EGF11–12 and the DSL domain residues interact to promote Notch trans-activation. In contrast, the molecular requirements for *cis* interactions are less clear cut, although the loss of both cis-inhibition and trans-activation, observed when a panel of Serrate DSL mutants were over expressed in the imaginal disc, suggest that the DSL region is involved in both [11]. Early reports proposed that the Abruptex region of Notch receptor (EGF24–29) might be involved in cis interactions [36]. A recent study showed that the Notch EGF10–12 region is required for cis-inhibition [37]. Although similar regions of receptor and ligand are implicated, it is impossible to predict whether or not a single molecular complex involving DSL and EGF11–12 underlies both types of interaction. NMR analysis and *in silico* docking of isolated receptor ligand fragments have suggested that two distinct complexes involving the same regions could form, however in the absence of more native-like flanking sequences, these data should be regarded as preliminary. More convincing data may come from the expression of full length Notch mutants and observation of their effects in an experimental system which can distinguish cis-inhibition and trans-activation.

### 4.1 Insights into Notch–ligand interaction: affinity vs. avidity

Although new insights into the Notch–ligand recognition event have been gained from the application of structural biology, the establishment of a quantitative assay is required to identify the affinity of different Notch ligand complexes. This is required to fine tune the design of fragments for co-crystallisation, to determine the contribution, if any, of flanking regions and post translational-modifications to the interaction, and to rationalise the contribution of affinity/avidity to the activation mechanism. A relatively weak *K*_D_ of 130 μM was measured for the calcium-dependent interaction of EGF11–14 Notch1 and DSL EGF-3 of Dll-1 in an SPR study [10]. In solid phase assays, mouse soluble Dll-1 produced from eukaryotic cells showed saturable binding at nM concentrations to larger fragments of Notch 1 and Notch 3 (N-terminus to EGF15) and a soluble Jagged1 construct gave a *K*_D(app)_ value of 0.7 nM suggesting that regions outside the EGF11–12 region may contribute to ligand binding [38,39]. However the ligand fragments were expressed as Fc fusions, so avidity effects may lie behind the very different *K*_D(app)_ values reported, compared to the studies performed with monomeric reagents. Similar ELISA based assays of fragments of *Drosophila* Notch and Delta suggested that the ligand-binding region of Notch also bound to the Abruptex region and a role of Abruptex in maintaining the inactive state of the receptor was proposed [40]. Comparative analysis of Fc vs. non-Fc-fused ligand constructs would establish the effect of avidity on the measured *K*_D(app)_, and pave the way for systematic analysis of the contribution of regions outside the recognition domains, and/or post-translational modifications to binding using ELISA and SPR solid phase assays. These data would greatly facilitate the design of receptor and ligand fragments with optimal binding affinity, which may prove suitable for co-crystallisation studies.

As described earlier the early steps leading to activation upon ligand binding are undefined. Is the receptor activated solely by mechanotransduction initiated by ligand endocytosis and/or do a series of conformational changes lead to an allosteric effect on S2 cleavage? Cell aggregation assays performed on Serrate DSL mutants demonstrated that F249A and R253A retained their ability to bind to Notch despite being defective in transactivation, suggesting that binding alone is not sufficient to initiate the signalling process [11]. These mutants would be prime candidates to test in quantitative binding assays, since native-like binding would suggest the involvement of these conserved residues in stabilising a conformation required for activation, rather than contributing to the stability of the complex. Interestingly two separate studies showed that N-terminal linkage of EGF10 reduced the ligand binding affinity of a receptor fragment encompassing EGF11–12 [10,27]. In the latter study, ligand binding could be rescued by the introduction of a calcium-binding mutation into EGF11 which uncouples the EGF10–11 interface. These data suggest that conformational changes in regions adjacent to EGF12 have the capacity to modulate ligand binding, but until these experiments are repeated using larger fragments of Notch, it is unclear if they have physiological relevance. Ligand-dependent clustering of Notch has been proposed to allow trans-regulatory complexes to withstand the mechanical pulling force generated by ligand endocytosis during the activation process suggesting that avidity effects may contribute to the mechanism of activation [41]. Atomic force microscopy on live cells showed that Notch signalling is linked to the adhesion force between cells expressing receptor and ligand [42]. The surface of S2 cells expressing Notch and Delta revealed marked differences with Notch-expressing cells displaying a topology of fibres whereas Delta expressing cells showed bumps. The authors predicted that Notch might exist as a monomer or oligomer, but proposed that Delta formed a multimer. On the basis of this it was postulated that Delta and Notch-expressing cells resemble ‘Velcro’. O-fut1 down-regulation by RNA interference produced a marked difference of surface topology and resulted in almost zero detachment force to Delta expressing cells. It is possible therefore that changes in glycosylation state could alter the conformation of the receptor/ligand and their ability to cluster which in turn would modulate binding and activation.

## 5 Notch–ligand therapeutics: where lies the specificity?

Consistent with its central role in metazoan cell–cell communication, dysregulation of Notch signalling leads to several genetic and acquired diseases. Several mutations were identified in receptors and ligands causing developmental disorders Alagille syndrome, Spondylocostal dysostosis, Tetrology of Fallot, aortic valve disease and adult-onset conditions such as CADASIL [43]. Notch signalling has also been found to be dysregulated in several cancers which make it a potential target for anti-cancer therapeutics [44–47]. The current strategies target key events in Notch signalling in the form of decoy soluble ligands and receptors, TACE inhibitors, γ-secretase inhibitors (GSIs), and transcription complex inhibitors. The GSIs have reached clinical trials, but they are not selective for different Notch receptors and are associated with significant levels of intestinal toxicity. With the availability of high resolution structures for regions involved in the activation process such as NRR, paralogue-specific antibodies are under development which are designed to block activation [48]. Wu et al., have recently shown that dual inhibition of Notch-1 and Notch-2 cause intestinal toxicity, whereas specifically targeting Notch-1 or Notch-2 with anti-NRR antibodies avoids toxicity [49]. These data suggest that therapeutic antibodies targeted against other regions of the receptor and ligand involved in binding and activation may also allow specific targeting of Notch/ligand paralogues.

## 6 Conclusions and perspective

It is perhaps surprising, given its known role in development and disease, that the Notch signalling pathway remains relatively poorly understood at a molecular level. However, recent years have seen the publication of high resolution structures for individual parts of the receptor, ligand, and transcriptional complex (Table 1), suggesting that a more complete molecular description is a realistic prospect. In future, combining biochemical and biophysical skills in the areas of protein purification, O-glycosylation, receptor endocytosis, and structural biology should allow a molecular dissection of the activation mechanism and its regulation by post-translational modifications and, as a consequence, offer new targets for cancer therapies.

## Figures and Tables

**Table 1:** Structures of Notch receptor and ligand fragments.

Molecule	Organism	PDB ID	Method
Ligands			
Jagged-1, domains DSL and EGFs 1–3	Human	2VJ2	X-ray (2.5 Å)
Jagged-1, exon 6	Human	2KB9	NMR
Notch extracellular domains			
Notch-1 EGFs 11–13	Human	1TOZ	NMR
Notch-1 EGFs 11–13	Human	2VJ3	X-ray (2.6 Å)
Notch-1 EGF 12	Synthetic	2RR0	NMR
Notch-1 EGF 12 with sugar modification	Synthetic	2RQZ	NMR
Notch-1 EGF 12 with O-fucosylation	Synthetic	2RR2	NMR
Notch-1 Lin12 module	Human	1PB5	NMR
Notch-2 negative regulatory region	Human	2OO4	X-ray (2.0 Å)
Notch1 negative regulatory region	Human	3ETO	X-ray (2.0 Å)
S1 cleaved Notch1 negative regulatory region	Human	3IO8	X-ray (3.2 Å)
Notch-1 NRR bound to fab of antagonist antibody	Human	3L95	X-ray (2.19 Å)
Notch extracellular domain	Fly	Ref. [28]	EM
Notch extracellular domain	Human	Ref. [28]	EM
Notch intracellular domains			
Notch ankyrin domain repeats 4–7	Fly	1OT8	X-ray (2.0 Å)
Notch-1 ankyrin domain	Mouse	1YMP	X-ray (2.2 Å)
Notch-1 ankyrin domain	Human	1YYH	X-ray (1.9 Å)
Notch-1 ankyrin domain	Human	2F8Y	X-ray (1.55 Å)
Notch-1 ankyrin domain mutant	Human	2HEO	X-ray (1.9 Å)
Notch-1 ankyrin domain	Mouse	2QC9	X-ray (2.35 Å)
Ankyrin domain	Synthetic	2ZGG	X-ray (2.0 Å)
Notch,CSL and MAML on HES-1 promoter	Human	2F8X	X-ray (3.25 Å)
CSL, Notch and Mastermind bound to DNA	Worm	2FO1	X-ray (3.12 Å)
CSL(Lag-1) bound to DNA with Lin-12 RAM peptide	Worm	3BRD	X-ray (2.21 Å)
Notch transcription complex on dual CSL sites	Human	3NBN	X-ray (3.45 Å)
